# Ebola virus mRNAs contain RNA structures that are critical for viral infection and targetable by antisense oligonucleotides

**DOI:** 10.64898/2026.09.04.749507

**Published:** 2026-09-08

**Authors:** Michelle Luo, Judith Olejnik, Kristina Meier, Tanja Hann, Elke Mühlberger, Anna Marie Pyle

## Abstract

Filoviruses, such as Ebola virus (EBOV), are highly pathogenic non-segmented negative-sense RNA viruses (nsNSVs) with limited therapeutic options. Filovirus RNA structures remain largely untapped due to the enhanced biosafety requirements for handling infectious virus. Here, we present the first in-cell secondary structure maps of four EBOV mRNAs (VP35, VP40, VP30, and VP24) using two orthogonal chemical probing approaches: SHAPE-MaP and fbDMS-MaP. We find that EBOV mRNA coding sequences (CDS) are highly structured, much like +ssRNA viruses, whereas untranslated regions (UTRs) are significantly less structured. This suggests that high CDS structure contents are general features of viral translation templates, and that nsNSVs have evolved separate regulatory function at the RNA structure level that extends beyond using distinct mRNAs and genomes. These structure maps are consistent with formation of mRNA 5′ hairpin structures during infection and reveal numerous additional RNA structures within the CDS, 3′ UTRs, and at CDS-UTR junctions. To assess functionality, we disrupted these structures with locked nucleic acid (LNA) antisense oligonucleotides. Disrupting the TSS hairpins in VP35, VP30, and VP24 decreased infection by >60%, indicating these mRNA structures are critical for infection. LNA targeting of the newly identified structures reduced EBOV infection by 31% to 88%, thereby linking RNA structural integrity to viral function. Synonymous mutation rates and covariation analysis provided evolutionary support across mammalian filoviruses for the functional RNA elements observed. Collectively, these results demonstrate EBOV mRNAs contain numerous conserved RNA motifs contributing to viral infection, and that these elements represent promising targets for development of pan-filoviral therapeutics.

**Importance:**

EBOV and related filoviruses pose a significant global health threat, yet our understanding of the RNA architectural mechanisms driving infection remains incomplete. Due to enhanced biosafety constraints, filovirus RNA structures have not been mapped in cells. Here, we experimentally map secondary structures of four EBOV mRNAs at biosafety level 4. We find that structure content is concentrated within protein coding regions, resembling the highly structured genomes of positive-sense RNA viruses, while untranslated regions are relatively unstructured. This separation of relative structural content reflects how translation and replication are delegated between mRNAs and genomes in negative-sense RNA viruses. We also show that these structures are critical for infection, with disruption reducing infection more than 80% in liver cells. Evolutionary analyses suggest a set of these regulatory elements are conserved across mammalian filoviruses. Broadly, this work expands the repertoire of filovirus regulatory elements, revealing new potential targets for developing pan-filoviral therapeutics.

## Full Text Availability

The license terms selected by the author(s) for this preprint version do not permit archiving in PMC. The full text is available from the preprint server.

